# The Plight of Parents/Caregivers of Children with Heart Disease in the Rural Areas of Namibia: A Problem of Coping

**DOI:** 10.5539/gjhs.v5n2p62

**Published:** 2012-12-04

**Authors:** Kristofina Amakali, Louis F. Small

**Affiliations:** 1 School of Nursing and Public Health, University of Namibia (UNAM), Windhoek, Namibia

**Keywords:** parents, caregivers, coping, care demands, children, heart disease, rural area

## Abstract

Providing care for a child with heart disease is a daunting task for any parent/caregiver, particularly for those living in poor conditions in rural Namibia. A qualitative, exploratory, phenomenological and contextual study was conducted to describe such parents’/caregivers’ experiences of providing care for a child with a heart disease. The study also examined the children's experiences of living with the burden of disease at home. The findings revealed experiences of emotional turmoil, disruptive social functioning and social relations, lack of support from the family, lack of organised forms of support from societal organisations, as well as experiences of low vitality among the children. These experiences together signify the overall poor coping by the parents/caregivers and the children. This paper presents the findings of a situational analysis of the experiences of caring for a child with heart disease and of living with heart disease.

## 1. Background

In 2005, the incidence of congenital heart disease (CHD) was reported to be eight out of 1000 live infants born worldwide (Opie, Bongani, & Mayosi, 2005). In Namibia, heart diseases contribute to about ten percent of all paediatric admissions to healthcare facilities. Congenital heart defects and rheumatic heart disease are the main causes of heart disease for these children and account for 60% and 40%, respectively. In Namibia, congenital heart defects account for nine percent of neonatal death. There are, therefore, many children under the age of 18 in that country who are currently in need of cardiac (heart) surgery (WHO, 2009; Du Toit, 2008).

Despite the intensity of the heart diseases, the majority of these children receive treatment as outpatients and their parents/caregivers are expected to cope with the demands of caring for these children at home. To provide the required care for a child with a heart disease requires vigilance on the part of the parents/caregivers. This assumes that they have the necessary knowledge, skills and financial means, as well as support systems in place, to prevent treatment-related setbacks and complications and to ensure quality of life for these children (Du Toit, 2008; Coovadia & Wittenberg, 2007; Beck & Wiencek-Kurek, 2007).

However, this assumption is not true for the majority of cases in Namibia. Many of these children live in rural areas and their parents/caregivers have a low level of education and, consequently, no secure sources of incomes, depending on subsistence agriculture only. Thus, as in any situation of poverty, parents are unable to provide the necessary care (Collins, Bronte-Tinkew, & Logan, 2008).Taking care of a child with heart disease then becomes a daunting task because of the complexity of such disease and the financial burden that the demands of care place on these parents.

## 2. Purpose of the Study

The purpose of the study was to explore and describe the lived experiences of parents/caregivers caring for children with heart disease, as well as the experiences of the children themselves who are living with heart disease. The intention was firstly to interpret the way the two parties cope with the situation at home, and secondly, to develop a healthcare programme to facilitate parents/caregivers’ coping with the demands of care at home.

## 3. Study Design and Methods

A qualitative, phenomenological and contextual study was conducted to explore and describe the parents’/caregivers’ experiences of caring for a child with heart disease and the children's experiences of living with heart disease. A sample of five multiple cases comprising a parent/caregiver and their child with heart disease living in a rural area was purposefully selected. The data were collected using in-depth interviews, observation of all participant categories and naïve drawings made by the child participants. The data were collected until saturation point was reached (Streubert-Speziale & Carpenter, 2007), and then analysed according to the Tesch method of qualitative data analysis (Creswell, 2008).

**Figure 1:**
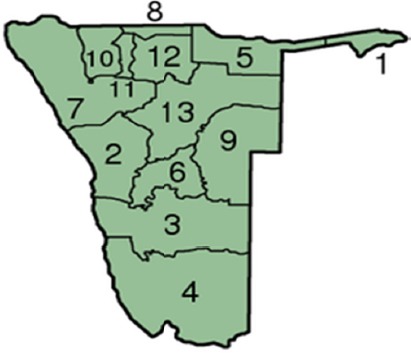
Map of Namibia esituated in regions 1, 10 and 11

The sampling criteria used for this study were the participants’ context and language of communication, which was the participants’ mother tongue. This was important in order for participants to discuss their experiences relating to their cultural and economic background and to be able to express themselves effectively (De Vos, Strydom, Fouche, & Delport, 2007; Burns & Grove, 2004; Patton, 2002). The participants were from rural areas in three different regions of Namibia and were of low socioeconomic status and living in households headed by a subsistence farmer. Accordingly, these criteria place the parents/caregivers at risk of poor coping (Collins et al., 2008). For the purposes of data collection, it was decided to select participants from different regions and cultural practices in Namibia in order to verify the assumption that socioeconomic factors affect coping experiences similarly across cultures in Namibia (Streubert-Speziale & Carpenter, 2007). In order for participants to be able to express themselves, the interviews were conducted in their mother tongue, except in cases where English was the only common language between the participant and the researcher. Figure 1 provides the details of the regions and residential areas of the study participants, while table 1 displays the demographic information on the participants.

**Table 1:** Demographic information on the participants

Multiple-Case	Participant	Age	Gender	Level of schooling	Diagnosis
1	Parent	38	F	Grade 10	
	Child	17	M	Grade 10	Ventricular septal defect
2	Parent	44	F	Grade 7	
	Child	12	F	Grade 8	Rheumatic heart disease
3	Parent	44	F	Grade 12	
	Child	12	M	Grade 7	Rheumatic heart disease
4	Parent	44	F	Grade 5	
	Child	12	M	Grade 6	Rheumatic heart disease
5	Caregiver	29	F	Grade 10	
	Child	12	F	Grade 7	Tetralogy-Fallot

## 3. The Findings

The findings of the study revealed negative experiences among the parents/caregivers and the children. Four themes were identified from the data analysis: The first theme is related to experiences of emotional challenges for both categories of participants, that is, parents/caregivers, on the one hand, and the children living with heart disease on the other. These emotional challenges are characterised as shock, disbelief and sadness over the child's diagnosis, fear for the death of the child and a sense of hopelessness in terms of caring for a child with heart disease.

The second theme is also related to both categories of participants and is concerned with the experiences of social dysfunction and lack of social relations owing to the demands that caring places on the parents and the impact that the disease may have on the child.

The third theme, which is of an economic nature, refers to the parents/caregivers only and is related to the experiences of financial difficulties in regard to providing care for the children concerned.

By contrast, the fourth theme refers to the children only, and relates to experiences of symptoms of limited physical functioning.

## 4. Discussion of Themes and the Literature Control

**Theme 1: The parents/caregivers and the children both experience emotional challenges**

As the literature shows, when a child's illness is diagnosed, the emotional challenges generally fall onto the parents or caregivers (Webb, 2009). Accordingly, the parent/caregiver participants talked movingly about their initial and continuing emotional responses to their child's illness.

**Figure 2:**
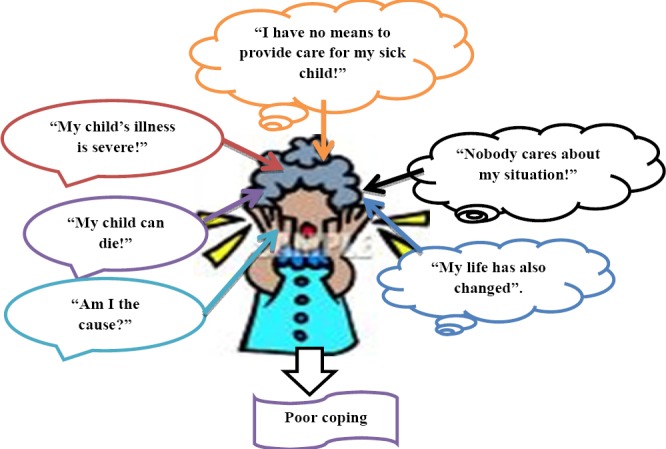
Illustration of the parents’/caregivers’ experience of challenges regarding caregiving for their children with heart disease – “Things fall apart!” (sourced from www.shutterstock.com)

**Figure 3:**
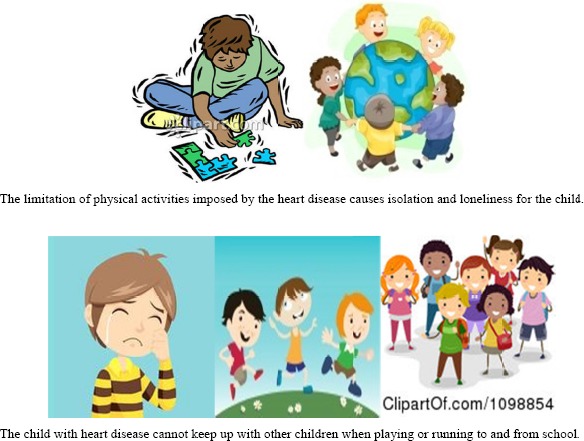
Illustration of the children's naïve drawings of their experiences of social isolation as a result of suffering from heart disease (sourced from www.shutterstock.com)

Some parents/caregivers reported that, at the initial diagnosis, the whole family experienced shock, presumably because of their perception that the heart is the organ that is vital to an individual's survival. This is evidenced by the following response by one of the participants.

“The heart is the one that keeps people alive. So I perceive the illness of my child as of severe degree. Therefore, I feel that my child can die anytime.”

It would appear that while parents/caregivers expect their children to contract the occasional minor childhood illness, they typically might not have considered that their children might develop heart disease, the serious nature of which compromises body functioning and requires adaptation, and which may not even have a cure. Therefore, as the literature has shown, parents/caregivers grieve the loss of their child's health, consequently implying heightened feelings of love towards their ill children, wanting to do things for them and spend time with them and provide all the care that they (the children) need (Webb, 2009; Ågren, 2008; Brosig, Pierucci & Leuthner, 2007; Deyirmenjian, Karam, & Salameh, 2005; Raina et al., 2005; Pinquart & Sörensen, 2003; Evangelista, Dracup, Doering, Westlake, Hamilton, & Fonarow, 2002).

As it is essential for both parents/caregivers and children to cope with the situation, Webb (2009) suggests that they should try to put a brave face on, as the child's own coping effort depends on that of the parents/caregivers.

Furthermore, the study found that parents/caregivers are not the only ones who manifest negative emotions and feelings about their children's condition. The observational data collected on the children also indicate that they too experience emotional challenges as a result of their illness. Therefore, although most children feel strong and invulnerable, the participants in this study felt inadequate. In concurrence with claims made by previous studies, the emotional challenges experienced by the children in this study are manifested in a range of emotional reactions, such as irritability and mood swings (Luzark, Jones, Slusher, Limbers, Burwinkle, & Varni, 2008; Ulvik, Henestad, Wentzel-Larsen, & Wahl, 2008; Deyirmenjian et al., 2005).

These children were thus less likely to accept the constraints of heart disease, as was reflected by either a simple non-response or a deflecting of the responses to the question or they simply stated that they felt their lives were no longer normal because they were sick and therefore they wished to be cured, as evidenced by the following statements.

“I feel bad because I am sick, and I feel bad because my life is not normal.”

Moreover, as the parents and caregivers experience negative feelings about their children's condition, they feel guilty when their children experience symptoms such as difficulty in breathing and exhaustion, which are both the result of heart disease. Therefore, instead of being empathetic, the parents’/caregivers’ emotional responses towards the children's conditions are more sympathetic, a phenomenon that calls for bereavement counselling directed at increasing the parents’/caregivers’ sense of self-control (Dracup et al., 2004).

Additionally, and as the existing literature suggests, parents/caregivers may employed a causal focus appraisal, blaming themselves for their children's condition. They alsoused their religious beliefs to explain why their children have acquired heart disease or even blamed themselves for having failed to do what they should have done for their children (Glanz, Rimer, & Viswanath, 2008; Forrester, 2008).

To this end, the literature still cautions that, while sympathy and empathy are important as well as nurturing for the child, overuse of these may result in parents/caregivers experiencing guilty feelings and self-blame and inadvertently reinforcing illness behaviours in the child (Van Tilburg et al., 2009).

Therefore, Webb (2009) cautions that while it is natural for parents and caregivers to look for a reason behind the diagnosis of the child's medical condition, viewing the illness as a form of punishment for wrongdoing or feeling that someone is to blame or displaying complete denial create maladaptation and poor coping. The tendency for parents to blame themselves for their child's condition may be attributed to a lack of knowledge of the illness, as well as not being knowledgeable about the resources that could be of assistance in their caregiving (Shu-Fan, Pei-Fan, & Kai-Sheng, 2008; Arafa, Zaher, EI-Dowaty, & Moneeb, 2007).

Furthermore, and in concurrence with the literature, when a diagnosis of heart disease is confirmed, not only do parents/caregivers lose healthy children, but they also lose self-confidence in their ability to meet the demands of caring for these children. The parents/caregivers in this study felt inadequate and hopeless and doubted their ability to care for their children at home. The following quotes attest to this (Cox et al., 2009; Van Tilburg et al., 2009; Ulvik et al., 2008; Lowes & Lyne, 2000).

“When I cannot afford the food that my child can tolerate because of the illness, I feel hopeless.”

It is obvious that the emotional challenges experienced by the parents/caregivers can translate into poor coping and, as a result, they may have a negative impact on the quality of care provided to the child (Chummun, Gospaul, & Lutchman, 2009).

However, the literature argues that these types of response to challenges tend to hold the parents/caregivers in a circle of concern, thereby reducing their chances of being proactive and engaging more with the circle of influences that could help them cope with their situation. Dwelling on negative perceptions results in a reduction in seeking positive influences and carrying out actions that can promote a sense of purpose in life, such as seeking information and connecting with others, which result in enhanced coping and positive health outcomes for the children as the beneficiaries of the caring effort (Vollman, LaMontagne, & Walston, 2009; Glanz et al., 2008; Covey, 2004). It is therefore recommended that these parents and caregivers be counselled and provided with appropriate information about the course of their child's illness. They should also be encouraged to carry out prescribed therapeutic and rehabilitative measures effectively, as these can help them to cope with the demands of care (George, 2008).

**Theme 2: The participants (parents/caregivers and the children) are living with disrupted social functioning and social relations**

In line with analogous studies, parents/caregivers told of their need to stay at home to supervise the sick child, which limits the degree to which they can engage in other social roles, restricts their opportunities for recreation and socialisation, and brings about changes in family life. In this regard, primary caregivers experienced a vicious circle of psychological preoccupation with the thoughts of the sick child and less meaningful fulfilment in other social roles (Arafa et al., 2008; Major, 2003).

In addition to being concerned about the effects that the demands of care have on their social lives, caregivers are also concerned that the disease will impact negatively on the child's education. They were concerned that their children would not be able to complete their education, as some of these children either have erratic school attendance, or cannot attend school at all, owing to ill health and frequent hospitalisation, as evidenced by one of the caregiver's responses:

“I wish he could attend school to be successful.”

As in the case of their parents/caregivers, and as the literature indicates, the children appeared to employ a motivational relevance appraisal as they indicated that being sick with heart disease deprives them of the opportunity to socialise and get an education, owing to frequent hospitalisation and a restriction of activities (Webb, 2009). The following quote, among many, bears testimony:

“Others are at school and I cannot go to school. Others do things like soccer but I cannot do it.”

In addition to the children's verbally expressed perception of deprivation of opportunities for socialisation, they also stated that they experience confinement, social isolation and loneliness as a result of being sick. Unlike other studies, however, which state that engagement in exercises and reading often moderate experiences of depression among heart patients (Dekker, Peden, Lennie, Schooler, & Moser, 2009; Ågren, 2008), these children indicated that they have difficulty with for example playing, which can also moderate emotional distress and loneliness.

In addition to parents being concerned about the potential loss of opportunities for social achievement by their children, the literature postulates that although caregiving is a normal part of being a parent of a young child, the caring role may take on an entirely different significance when a child experiences limitations in self-care functions, such as feeding, bathing, dressing or potential long-term dependence, which far exceed the usual needs for childcare (Raina et al., 2005). There is evidence from this study that the parents/caregivers were overwhelmingly concerned that the incapacitating effects of the heart disease on the child could result in the child's inability to contribute to the family system, then and in the future, because these children can could longer do household chores or contribute to the family's wellbeing. In conclusion, this theme helped to show that the caring role imposes extra responsibilities on parents/caregivers and negatively affects the children's chances for socio-economic achievement. Moreover, the demands for care impose an economic burden on the families.

**Theme 3: The parents/caregivers experience a lack support from their families in addition to a lack of organised support from societal organisations**

The revelations of this study indicate that parents experience role overload, a lack of emotional support and a lack of financial resources. The parents/caregivers indicated that they are in dire need of “hands-on” assistance in performing the instrumental tasks of care for their children, items such as mattresses and pillows designed to ease breathing difficulties as well as financial assistance for both home-based care and healthcare services. Furthermore, the majority of parent/caregivers indicated that they need to understand the nature of their child's illness and the skills they need in order to carry out the instrumental tasks of caregiving at home. It was noted that very little support is available from either the participants’ families or societal organisations (Forrester, 2008; Glanz et al., 2008).

As can be inferred from parents’/caregivers’ assertions, and supported by parallel studies, a lack of support from families and societal organisations seems to amplify the experience of poor coping among parents/caregivers. Their relatives and friends fail to provide them with the on-going support that they hoped for. As a result, they felt betrayed by their families, friends and society, and their expectations of support come to nothing. Worst of all is the fact that most of the cases that participated in this study are single-parent, female-headed households, a factor that exacerbates poverty and increases vulnerability to financial crises for these families and, in turn, results in poor coping with the demands of care (Stajduhar, Leigh Martin, Barwish, & Fyle, 2008; Brosig et al., 2007; Deyirmenjian et al., 2005). Therefore, one of the first effects of financial difficulty is related to the inability to provide a cardiac diet for the children concerned, as these caregivers are not financially able to buy the types of food that are recommended for a patient with heart disease, most of which are not routinely available at the household level, but have to be bought from the market, which they simply cannot afford.

Therefore, the recommendations with regard to the [cardiac] diet (which is nutritious but less risky for the heart, such as fruit and vegetables) are not followed To make things worse, any efforts by the parents/caregivers to obtain the foods for the children from the community-based government initiatives for food relief for the needy have proven fruitless because the needs of these children (with heart disease) are not catered for by such initiatives. As a result of financial constraints, parents/caregivers admitted that they only give the food that is available at the household level, such as cow's milk, dry spinach served with mahangu pap, fish and chicken, or, rarely, a pumpkin if they have a good harvest. So a typical meal for a child with heart disease from a low socioeconomic background in Namibia would be made from that short-list of food and the child has to eat that for every meal. The following statement is evidence of this:

“The foods that I have to give to the child are not available at home. You need to buy them, but there is no money because I do not earn money.”

It is obvious that a diet poor in nutrition, coupled with low metabolism, which is a physiological factor in heart disease, are causes of failure to thrive among these children, as all of the children in this study were low weight for their age (Menon & Poskitt, 1985).

Given all the hurdles these parents and caregivers experience, all of them appealed for the provision of food supplements by the government as an integral part of the health services approach to children with heart disease. As these parents and caregivers experience a financial burden at a household level, it is obvious that financial hardship also manifests in difficulties obtaining transport to facilitate follow-up treatment and in the cost of treatment.

Hence, the second aspect of financial difficulty relates to the expenses involved in travelling to the place where the children receive follow-up treatment. Access to health care for children with heart disease in the Namibian context involves being able to access the locations where the required specialised health services are offered. Although these children are eligible for treatment as state patients, their parents/caregivers nonetheless have to find the money to cover the transport. The caregivers indicated that, in some instances, as none of their family is employed, they have to borrow money from their neighbours, consequently accumulating debt.

*“Because of travelling to hospital, I spent all the money I had. So I had to borrow money from other people. Now I am indebted to other people”*.

As these parents/caregivers sometimes cannot afford the costs incurred, children are at risk of missing their medical consultation and, as a result, may experience high morbidity and even mortality. Lack of financial resources for travelling to and from the hospital is reported to translate into further experience of disruptive social functioning, as in some cases the parent/caregiver and the child have to stay longer at the clinic or hospital so that the child can attend at least two consecutive follow-up treatments before they go home. Even worse, a lack of financial resources may often result in a delayed treatment process, with the child suffering for a more prolonged period and increased demands for care being placed on the parents/caregivers, which in turn results in disruptive social functioning and negative emotional experiences. There is no doubt that such a vicious circle of negative experiences will ultimately result in poor coping on the part of parents/caregivers. Indeed, as the literature proposes, a lack of financial means can impede access to the healthcare services that will optimise health, ability and the general development of these children (Webb, 2009; Dekker et al., 2009; Beck & Wiencek-Kurek, 2007).

In conclusion, the findings provide evidence of extreme poverty at the household level as characterised by a lack of access to the prescribed cardiac diet and the basic facilities for easy care at home. Thus, financial problems are one of the most important aspects of this study, because all the dimensions of poor coping seem to be rooted in a lack of financial means to facilitate coping with the demands of care at home.

In addition to the resources that are necessary to facilitate provision of care at home and access to treatment facilities, the possession of relevant knowledge by parents/caregivers is a valuable asset that may counterbalance the difficulties experienced in caring for the child at home.

Nevertheless, the findings of this study indicate that the majority of the parents/caregivers lacked knowledge of the child's disease and the potential treatment outcomes. Therefore, they need assistance from someone outside the family to counsel the child in order for he, or she to understand the nature of the illness. This implies that they were not given sufficient information about their children's condition and care. Indeed, some of the parents/caregivers asserted that they had high expectations of their children even when the child's condition could not be surgically corrected, but a case for symptomatic care only.

“I wish he could attend school to be successful; if there could be a special school near the hospital for him to attend school because I wish him to succeed at school.”

In concurrence with the existing literature, it is evident that the communication of medical information and information about care continuity to the parents/caregivers of children with heart disease is problematic, a state which of course influences the manner in which the facts of the illness (diagnosis, symptomatic care and treatment outcomes) are received or borne.

It is obvious that healthcare providers give parents very little honest information about the child's illness and treatment, which can exacerbate these parents’/caregivers’ lack of confidence, and their inability to deal with what is perceived as “the difficult questions” from the children. It also interrupts the continuity of care that they (parents/caregivers) give to their children (Bugge, Helseth & Darbyshire, 2009; Paul, 2008; Dickstein et al., 2008; Forrester, 2008; Lainšćak, Cleland, & Lenzen, 2007; Helseth & Ulfsaet, 2005; Strömberg, 2005).

**Theme 4: The children experience decreased vitality**

The fourth theme that was identified and is exclusive to the children relates to the children's personal appraisal of their quality of life as a result of having heart disease. In this regard, and consistent with the literature, the children presented self-reported experiences of low physical vitality and other physical symptoms, such as exhaustion after physical activities, chest pain, shortness of breath, insomnia, feeling bloated and nauseous, to mention just a few. These are, of course, the adverse effects of heart disease on the children's physiology (Heo, Lennie, Okoli, & Morser, 2009; Dekker et al., 2009; Davidson, Cockburn, & Newton, 2008; Bennet, Cordes, Westmoreland, Castro, & Donnely, 2000).

“I do not sleep restful at night because I feel chest pain.”

“I am troubled by the symptoms. When walking from school, I get tired and I have to sit down.”

“I also do not like some of the experiences I have because of my illness such as stomach distension & discomfort and getting tired easily especially after running.”

In addition, the inferences of experiences of physical dysfunction were made based on the fact that, by the time of interview, some of the children were no longer in school owing to the physical limitations resulting from heart disease.

Although the physiological effects of the disease are confined to the child, the parents and caregivers supported the children's responses and explained that the children experience physical discomfort as characterised by exhaustion after physical activity and an inability to perform routine household chores or to walk to school. In some instances, the child even had to be escorted to and from school, all of which signal more demands for care on the part of the parents/caregivers (Blinderman, Homel, Billings, Portenoy, & Tennstedt, 2008).

In conclusion, unlike the claims of other studies, which place more emphasis on the experiences of psychological dysfunction, the experiences of physical dysfunction were alluded to more by the children in this study. In order to summarise the findings, the parents’/caregivers’ and children's experiences were related to the framework, the Transactional Model of Stress and Coping, as well as Kübler-Ross's stages of grief approach.

According to the Transactional Model of Stress and Coping, in response to a stressful situation, primary and secondary appraisal and coping methods are employed and, as a result, coping outcomes may be produced (Glanz et al., 2008).

*In accordance with the notion of primary appraisal*, the parents and caregivers perceived the condition of their children as being severe and life-threatening. Subsequently, they perceived a heightened vulnerability in their children, which according to them is an insurmountable challenge which can affect every dimensions of their life (Glanz et al., 2008). Therefore, according to Kübler-Ross's stages-of-grief approach (in Webb, 2009), the majority of the parents/caregivers were in an anger stage and were overwhelmed by depression, as signified by a sense of hopelessness. None of them expressed constructive acceptance of the child's condition.

Furthermore, the parents/caregivers employed a causal focus appraisal in terms of who was responsible for their child's plight. In addition, the parents/caregivers perceived the demands of care as being onerous and taxing; accordingly, they made an appeal for assistance to enable them to cope with the demands of care. The children with heart disease, on the other hand, employed a motivational relevance appraisal, thus regarding their illness as having major negative effects on their personal goals. In both cases, a heightened perception of vulnerability generated a sense of inadequacy and poor coping.

*In accordance with the notion of secondary appraisal*, the parents/caregivers of children with heart disease from rural areas of Namibia perceive they are unable to manage their emotional reactions. As a result, they feel inadequate and they do not believe that they have the resources (materials and social support) to facilitate the care that is necessary for their children or the ability to perform the health promotion activities necessary to improve children's quality of life. Therefore, they cope poorly with the demands of care at home. Moreover, the parents/caregivers experience problems with harmonising the responsibility for providing care with their other social responsibilities. As a result, during the research they asserted that they were not able to cope with the demands of care.

*Coping:* The parents/caregivers of children with heart disease engage less with neither of the coping methods, which resulted in them feeling inadequate and coping ineffectively.

**Figure 4:**
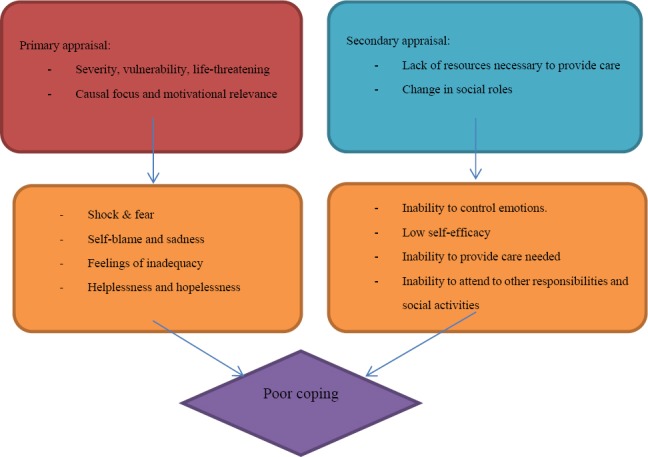
The parents/caregivers and the children's appraisals of their experiences

*Coping outcomes:* As the parents/caregivers of children with heart disease cannot employ constructive coping methods, they experience a negative self-concept as a result of their lack of coping skills. This compromises the quality of care and the quality of life for the children concerned. Both primary and secondary appraisals of the situation resulted in poor coping among parents and caregivers, as illustrated in figure 4.

The way in which challenges are experienced depends on how they are perceived and appraised by the person involved. A given challenge may be perceived differently by different people. It is therefore perceptions that determine the behaviours for coping and health outcomes (Glanz et al., 2008).

## 5. Conclusions

In conclusion, the findings of this study provide strong evidence of poor coping with the demands of care at home by the parents/caregivers. These findings are manifest in the reported experiences of emotional challenges, and the disruptive social functioning and social relations by both parents/caregivers and children. In addition, experiences of financial difficulties, lack of resources, lack of required knowledge by parents/caregivers and the decreased vitality experienced by the children are strongly supported by the literature.

The result is a vicious circle, in terms of which the children's unpleasant experiences increase the demands for care, consequently intensifying their parents’ emotional reactions and subsequently resulting in poor coping. It is therefore suggested that these parents/caregivers, as well as the families, need appropriate support to mitigate their negative experiences, provide appropriate care and facilitate positive health outcomes for the children concerned. By implication, poor coping with the demands of care means that these parents/caregivers require professional intervention in the form of a multicomponent programme of care, driven by the needs of the child and the caregivers, to help them cope. As a result of the findings of this study, a sustained home-based healthcare programme to empower parents/caregivers in coping with the demands of care at home was conceptualised

### Limitations of the Study

The data may have reflected only the perspectives that the respondents felt the researcher should hear or that are socially acceptable, rather than voicing their genuine experiences. In addition, as the data were filtered through the interviewer, the interpretation may be limited to the researcher's interpretation, resulting in limitations in the data. Furthermore, as is the nature of the findings of qualitative research, it may not be easy to generalise the conclusions from this study to others owing to the small sample size and the non-probability sampling method that were used in this study.

### Recommendations

Following from this research, key recommendations were made with regard to the clinical practice setting, health and social services and welfare, as well for future research. Healthcare providers should implement contextualised interventions to enable parents/caregivers to provide quality palliative care at home. A home-based healthcare programme in support of the parents/caregivers of these children should be developed, the implementation of which should be mainstreamed in healthcare programmes at the district level of healthcare delivery. In conclusion, social welfare services should be extended to the economically vulnerable families of children diagnosed with heart disease.
